# Investigating Dynamic Molecular Events in Melanoma Cell Nucleus During Photodynamic Therapy by SERS

**DOI:** 10.3389/fchem.2018.00665

**Published:** 2019-01-28

**Authors:** Jing Yue, Lijia Liang, Yanting Shen, Xin Guan, Jing Zhang, Zhiyuan Li, Rong Deng, Shuping Xu, Chongyang Liang, Wei Shi, Weiqing Xu

**Affiliations:** ^1^State Key Laboratory of Supramolecular Structure and Materials, Institute of Theoretical Chemistry, Jilin University, Changchun, China; ^2^Institute of Frontier Medical Science, Jilin University, Changchun, China; ^3^Key Lab for Molecular Enzymology and Engineering of Ministry of Education, Jilin University, Changchun, China

**Keywords:** Ce6, cell nucleus, real-time, photodynamic therapy, surface-enhanced Raman spectroscopy

## Abstract

Photodynamic therapy (PDT) involves the uptake of photosensitizers by cancer cells and the irradiation of a light with a specific wavelength to trigger a series of photochemical reactions based on the generation of reactive oxygen, leading to cancer cell death. PDT has been widely used in various fields of biomedicine. However, the molecular events of the cancer cell nucleus during the PDT process are still unclear. In this work, a nuclear-targeted gold nanorod Raman nanoprobe combined with surface-enhanced Raman scattering spectroscopy (SERS) was exploited to investigate the dynamic intranuclear molecular changes of B16 cells (a murine melanoma cell line) treated with a photosensitizer (Chlorin e6) and the specific light (650 nm). The SERS spectra of the cell nucleus during the PDT treatment were recorded *in situ* and the spectroscopic analysis of the dynamics of the nucleus uncovered two main events in the therapeutic process: the protein degradation and the DNA fragmentation. We expect that these findings are of vital significance in having a better understanding of the PDT mechanism acting on the cancer cell nucleus and can further help us to design and develop more effective therapeutic platforms and methods.

## Introduction

Over the last few decades, photodynamic therapy (PDT) has emerged as a new and significant therapeutic strategy for various diseases especially in cancer therapy and has been approved for cancer treatments in the clinic (Voon et al., 2014; Han et al., 2015; Sun et al., 2017). Compared with conventional cancer therapy strategies, PDT possesses noticeable merits including high controllability, target selectivity, localized damage and weak side effects. The treatment involves three key components: photosensitizer (PS), light and reactive oxygen species (ROS). The PSs used in PDT are usually non-invasive without light irradiation (Agostinis et al., 2011; Wang et al., 2013). Once there is a light with the appropriate wavelength, ROS especially singlet oxygen will generate, which can induce tumor death (Fan et al., 2016) since excess ROS can damage crucial biological macromolecules, including proteins, lipids, nucleic acids, and carbohydrates(Liu et al., 2012; Abrahamse and Hamblin, 2016). Also, ROS can induce a series of stress reactions if generated in high amounts. In early stages, the cells will activate the survival mechanism for self-defense or repair from injury. However, if the stress is so severe that cells can't protect themselves, the activation of death mechanisms will begin (Soriano et al., 2017). How cancer cells respond to the stressing situation depends on the cell types, the PS types and the level of ROS. In order to have deep understanding on the mechanisms of the PDT-induced cell death, many strategies have been used. Among them, cell viability tests (MTT and WST-1, etc.) and flow cytometry are the most common-used methods for PDT effect estimation in most literatures (Hodgkinson et al., 2017; Veloso et al., 2017; Yang et al., 2017). For instance, Soriano et al. used the MTT assay, Hoechst staining and nuclear morphology analysis to compare the photodynamic effects of two different PSs on non-tumoral and tumoral breast epithelial cell lines (Soriano et al., 2017). They found that most tumoral cells died from necrosis or apoptosis while non-tumoral cells died from necrosis mostly. These methods can only tell us the final therapeutic results by the ratio of living/dead cells, however, they can't provide any information about how PDT induced cell death. Currently, the response mechanisms of cells exposed to PDT have many different versions but are crucial either for learning the medical PDT therapeutic strategy or for understanding the cell self-defense process.

Surface-enhanced Raman spectroscopy (SERS) is a nearly non-invasive and powerful label-free analysis tool that can provide detailed fingerprint spectral information of cells, tissues and can achieve detection *in situ* (Kneipp et al., 2008; Qian and Nie, 2008; Kuku et al., 2017; Laing et al., 2017). In addition, SERS has inherent advantages of high sensitivity and real-time monitoring of complex and dynamic changes of analytes, which make it appropriate in multiplex biological processes (Kang et al., 2014; Ali et al., 2016; Kircher, 2016). On account of these superiorities, SERS has been widely used for *in situ* exploration of the structural information of intracellular molecules, as well as the dynamic changes of cells in response to some external stimuli, such as photo treatments and chemical drugs (Cialla-May et al., 2017; Kairdolf et al., 2017; Zheng et al., 2018). In previous work, we only found one paper reported by da Silva et al. (Veloso et al., 2017) who employed the direct SERS strategy to investigate cancer cell death caused by PDT. However, they adopted a destructive sample pre-treatment process in which all groups of the PDT-treated cells had been frozen in liquid nitrogen and then ground and stirred to obtain the liquid and homogeneous solutions for SERS detections. This pre-treatment fully destructed the cell framework and functional domains. While, SERS is sensitive to the molecular vibration, both the molecular structure and the localized environment can affect the obtained SERS signals and final results analysis. Additionally, although analyzing the changes of molecular information after the PDT treatment can provide some information about treatment effect, tracing the dynamic molecular events of the cell during the PDT treatment process is much more significant for understanding response mechanisms.

As the control center of cells, the cell nucleus plays important roles in metabolism, growth and differentiation. It is also the main site of genetic materials. A strategy for the *in-situ* SERS detection of cell nuclei has been developed, in which the plasmon-based nanoparticles are required to pre-incubate with cells and the Raman signals of intracellular components closely adjacent to these nanoparticles can be measured (Oyelere et al., 2007; Xie et al., 2009; Huefner et al., 2013). This *in situ* intranuclear SERS exploration method provides new access for the deeper study of cell biophysical processes from the cell nucleus aspect. Also, it provides a possible way to disclose the intracellular response toward external stimuli, particularly during cancer treatments (Austin et al., 2013; Liang et al., 2015; Deng et al., 2017; Shen et al., 2018).

**Figure 1:**
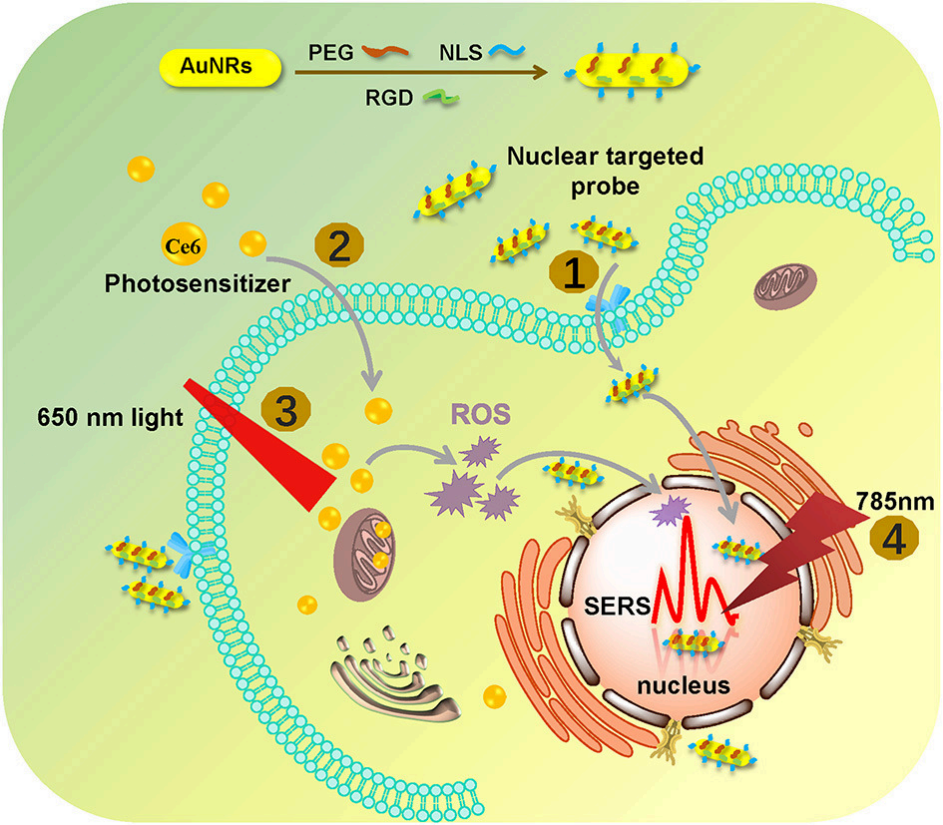
The procedures of monitoring the changes of the nuclear signal after photodynamic therapy by SERS spectroscopy with the AuNR-based nucleus-targeted nanoprobes. ①-④ correspond to the pre-loading of the targeting nanoprobes to the cell nucleus, the internalization of PS in the cell, the irradiation of Ce6 to produce ROS, and the SERS measurement of a cell nucleus.

In this work, by using the SERS technique combined with a nuclear-targeted gold nanorods (AuNRs) probe, we tracked the dynamics of the nucleus during the PDT treatment (as shown in Figure 1). A murine melanoma cell line (B16 cell) was selected as a proof of concept to evaluate its response behaviors during the PDT treatment. First, we modified the partial surface of AuNRs with the targeting peptides (cancer cell-specific targeted peptide and nuclear localization signal peptide) which can specially identify cancer cells and then deliver these nanoprobes to the nucleus accurately (①). Then Chlorin e6 (Ce6) as the PS was used for the PDT treatment of B16 cells (②), irradiated with a 650 nm light (③). Finally, the *in situ* SERS spectra of the nucleus during PDT treatment were recorded (④) and analyzed. This work mainly focused on exploring how the biomolecules of a cancer cell nucleus respond to PDT treatment by SERS spectroscopy, which is helpful for better understanding the PDT mechanism and further developing effective therapeutic approaches. The novelty of this work can be summarized as two aspects: (1) this is the first time to explore the acting mechanism of PDT on a cell nucleus based on the spectral information on nuclear components, and (2) it also the first one to monitor PDT with SERS *in situ*.

## Experimental

### Fabrication of Nuclear-Targeted Nanoprobes

First of all, AuNRs with a plasmonic absorption maximum of 753 nm (aspect ratio of about 4.4) which were stabilized by cetyltrimethylammonium bromide (CTAB) were synthesized. The details were shown in Supporting information. Ultraviolet–visible (UV–vis) spectroscopy, transmission electron microscopy (TEM) and dynamic light scattering (DLS) spectroscopy were employed to measure the plasmonic property, size, morphology, and zeta potential of the obtained AuNRs.

Then, AuNRs were modified by methoxy poly(ethylene glycol)-thiol (mPEG-SH) (MW = 5,000), nuclear localization signal (NLS) peptide, and a cancer-cell-specific targeted peptide (RGD) in one step. To quantify the number of peptides on a single AuNR, the calibration curve from the standards and the fluorescence of free FITC-labeled NLS before and after the reaction with AuNRs were required, as shown in Figure S1. According to the calibration curve (Figure S1b), the average number of NLS on a single AuNR is calculated as about 1100. The RGD on the surface of AuNRs was kept as the same dose ratio as NLS.

### Location of Nanoprobes in Nucleus

In order to confirm the targeting ability of the prepared nanoprobes, the cells grown on a slide glass for 24 h were incubated with fluorescein isothiocyanate (FITC)-labeled nuclear-targeted nanoprobes AuNRs-PEG-NLS-RGD (0.1 nM) prepared by linking FITC-tagged NLS (5.0 mM, 12 μL) on the surface of AuNRs (1.34 nM, 10 mL) for 24 h and next stained by Hoechst 33342 (excited by a 405 nm Laser, 10 μg/mL) for 15 min to highlight the cell nucleus. After that, a FV1000 confocal fluorescence microscope (Olympus) was used to confirm the location of the prepared nanoprobes.

In addition, the specific targeting effects of AuNRs-PEG-NLS-RGD were also evidenced by a self-built platform integrated with both fluorescence microscope (IX71, Olympus) and dark-field microscope (Olympus).

### Cell Culture

Murine melanoma cell line (B16 cells) were grown in Roswell Park Memorial Institute 1640 medium (RPMI 1640, Invitrogen) supplemented with 10% fetal bovine serum (FBS). The cells were maintained at 37°C in a humidified environment with 5% of CO_2_.

### Photosensitizer (Ce6) Preparation and Treatment of B16 Cells

In the dark, the photosensitizer Ce6 was dissolved in PBS in which oxygen was removed and we achieved a Ce6 solution with a concentration of 12 μM. B16 cells that had been planted on Quartz coverslips were cultured with the nanoprobes for 12 h, and then they were treated with the prepared Ce6 solution for 12 h. After that, cells were irradiated with an LED lamp (650 nm) at an energy density of 18 mW/cm^2^ for different lengths of time (0 to 5 min). Then cells were washed three times with phosphate-buffered saline (PBS) and fixed by 4% paraformaldehyde for next use.

### *In vitro* Cytotoxicity

*In vitro* cytotoxicity of AuNRs-PEG-NLS-RGD and Ce6 were assessed by the WST-1 (2-(4-Iodophenyl)-3-(4-nitrophenyl)-5-(2,4-disulfophenyl)-2H-tetrazolium, monosodium salt) assay. B16 cells were firstly grown in two 96-well plates in the RPMI Medium 1640 (1640, Thermo Fisher Scientific) containing 10% fetal bovine serum (FBS) at 37°C in a 5% CO_2_ incubator for 24 h. Then B16 cells in one 96-well plate were incubated with fresh culture medium containing 0.1 nM of AuNRs-based nanoprobes for another 24 h, and another 96-well was incubated with fresh culture medium containing different concentrations of Ce6 for the second 24 h. Afterwards, we added 20 μL of the WST-1 solution into each well and incubated them for 2 h continuously. At last, the absorption intensity of each well was measured at 450 nm by a microplate reader (Tecan Sunrise). We used B16 cells incubated with the standard cell culture medium for cell viability evaluation as the control group.

### Intracellular ROS Generation Assay

In order to detect the PDT-induced intracellular ROS, a 2, 7-dichlorofuorescin diacetate (DCFH-DA) probe was employed. DCFH-DA probe (10 μM) can monitor the generation of the intracellular ROS owing to a fluorescent turn-on chromogenic reaction from DCFH-DA to dichlorofluorescein (DCF) after undergoing intracellular deacetylation upon ROS-mediated oxidation. The B16 cells cultured in a Petri dish (1 × 10^5^) were co-cultured with nanoprobes (0.1 nM) and Ce6 (1.2 μM) for 12 h. After removing unbound NPs with PBS, the cells were incubated with 10 μM DCFH-DA at 37°C for 30 min. After that, cells were exposed to a 650 nm lamp (18 mW/cm^2^) for 1 min. Finally, the fluorescence of DCF was measured at 488 nm by using confocal fluorescence microscope.

### Apoptosis/Necrosis Assay

To further illustrate that PDT mediated the apoptosis of B16 cells, apoptosis/necrosis assay was applied by the flow cytometry combined with the Annexin V-APC/ 7-aminoactinomycinD (7-AAD). Annexin-VPC was used to differentiate apoptotic cells because of its strong affinity to phosphatidylserine serine on the outer membrane of apoptotic cells. And 7-AAD is generally excluded from live cells. After cultured for 24 h, cells were cultured with AuNRs-PEG-NLS-RGD and Ce6 for 12 h. After PDT, the cells in the suspension and the petri dish were collected. Finally, 100 μL of cells were stained with the Annexin V-APC for 7 min, 7-aminoactinomycinD (7-AAD) for 3 min and then 400 μL of PBS was added. Finally, the suspension was measured by the FACSCalibur (BD Biosciences, USA).

### SERS Detection of B16 Cell Nucleus

As mentioned above, B16 cells cultured already on quartz coverslips for 24 h were incubated with fresh culture medium containing of 0.1 nM nanoprobes and 1.2 μM Ce6 for another 12 h. Then cells were irradiated with the LED lamp (650 nm) at an energy density of 18 mW/cm^2^ for 0, 1, 3, and 5 min, respectively. After treatment, they were washed three times with PBS, fixed with 4% paraformaldehyde for 20 min, and then sealed for SERS detections. SERS detections were performed by a confocal Raman system (LabRAM Aramis, Horiba Jobin Yvon) with a 785 nm laser as the excitation source. The laser (with about 20 mW power on the sample) was directed into a microscope and focused on the sample by a 50 × 0.75 NA objective lens. All spectrums were obtained in a 30 s collection time with two accumulations. And the data was analyzed using NGSLabSpec and Origin 9.0.

## Results and Discussion

### Synthesis of Nuclear-Targeted Nanoprobes

In order to obtain molecular fingerprint information of the cell nucleus by SERS spectroscopy, a plasmon-based, nuclear-targeted nanoprobe is required and its SERS enhancement capacity should be high enough to achieve high-quality intranuclear spectra. Here, a targeting AuNR-based nanoprobe with the surface functionalization of RGD and NLS was prepared. Firstly, AuNRs were synthesized according to the seed-mediated growth method (Nikoobakht and El-Sayed, 2003; Sau and Murphy, 2004). To prevent aggregation and further improve the biocompatibility of the nanoprobes, mPEG-SH was conjugated to the surface of AuNRs. Next, RGD (CGGGPKKKRKGC) and NLS (GGVKRKKKPGGC) *via* the covalent linking between gold and the thiol group of cysteine (bold in the peptide sequence of NLS and RGD) to enrich the nanoprobe with the cancer cell selectivity and the nuclear-targeted ability. RGD can selectively recognize cancer cells *via* binding with the α*v*β*6* or α*v* integrins on the cell surface. NLS has a crucial sequence (KRKKK), which can deliver the nanoprobes to the cell nucleus through the nuclear pores.

**Figure 2:**
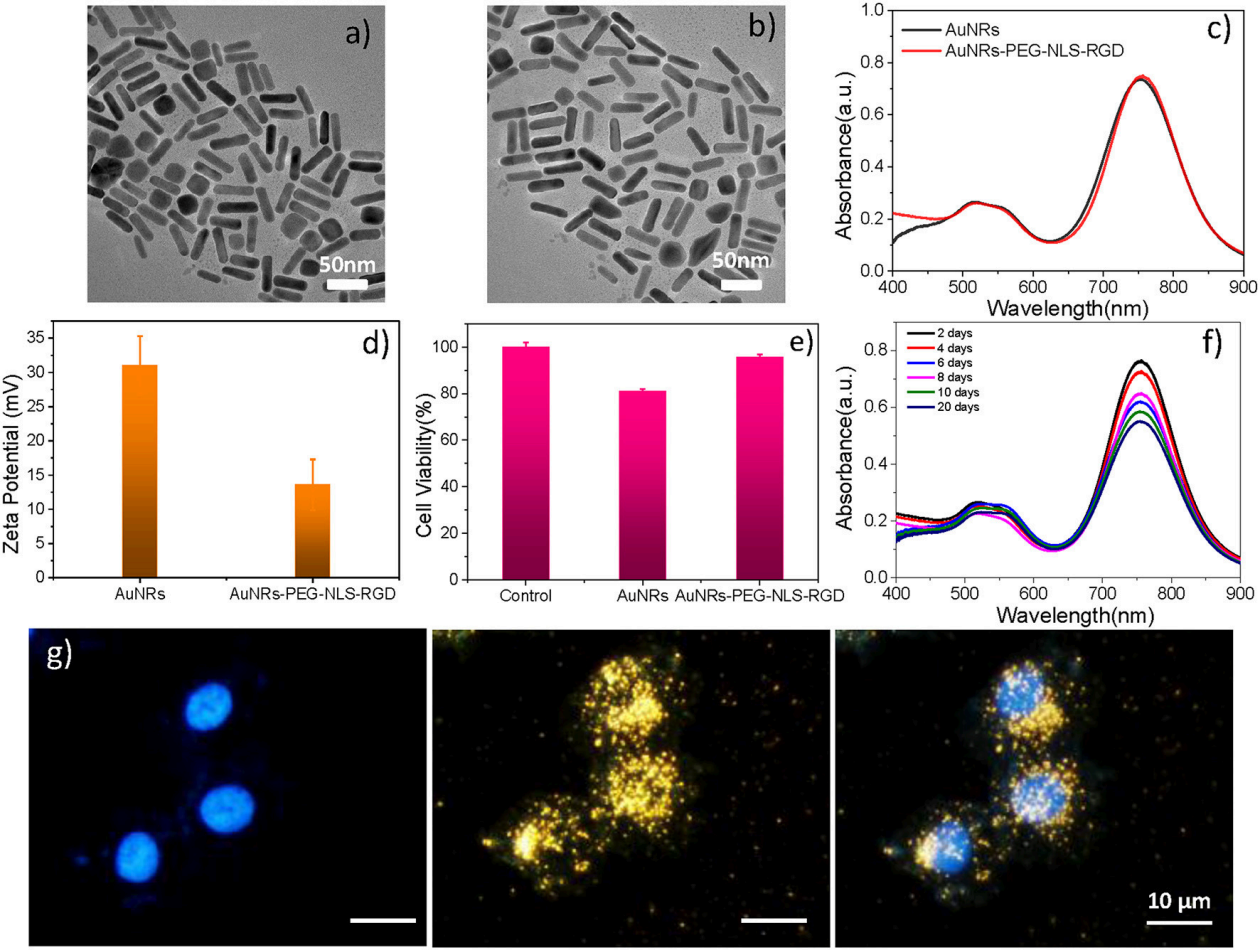
A TEM image of **(a)** AuNRs and **(b)** AuNRs-PEG-NLS-RGD. **(c)** UV–vis spectra of AuNRs and AuNRs-PEG-NLS-RGD. **(d)** Zeta potential of AuNRs and AuNRs-PEG-NLS-RGD. **(e)** Cell viabilities of B16 cells incubated with 0.1 nM of AuNRs and AuNRs-PEG-NLS-RGD for 24 h. **(f)** Uv-vis spectra of AuNRs-PEG-NLS-RGD nanoprobes after storage at 4°C for 20 days. **(g)** Fluorescent, dark-field and merged images of B16 cells incubated with AuNRs-PEG-NLS-RGD for 12 h from left to right.

As shown in Figure 2a, the AuNRs with an aspect ratio of about 4.4 (40 × 9 nm) were obtained. After surface modifications, the AuNR-based nuclear targeting nanoprobe (Figure 2b) keeps monodisperse and the size has no notable change compared with the bare AuNRs. Their plasmonic bands red shifts from 753 to 756 nm when AuNRs modifying with PEG, NLS and RGD (Figure 2c). And the zeta potential of AuNRs also show a decrease of 17 mV (from 31 to 14 mV, Figure 2d). In addition, considering that the AuNRs might affect cell metabolism and proliferation, the cell incubation concentration of nanoprobes was evaluated by the WST-1 assay, which is as low as 0.1 nM, indicating almost no toxicity to B16 cells (Figure 2e). Moreover, we further proved that the nanoprobe has acceptable stability after storage at 4°C for 20 days (Figure 2f) through Uv-vis spectroscopy. The decrease of absorbance might result from the colloidal deposition of a small amount of the AuNRs.

Targeting the AuNRs-based nanoprobes to purpose positions is the precondition for exploring the information of the intranuclear components. Our previous studies have demonstrated the targeting ability of the AuNRs-based nanoprobes in cancer cells [Soma gastric cancer cell: SGC-7901 (Liang et al., 2015) and liver cancer cell: HepG2 (Deng et al., 2017; Shen et al., 2018)] through dark-field and fluorescence images (Liang et al., 2015), high-resolution three-dimensional (3D) images, and bio-TEM images (Shen et al., 2018). Here, to prove the targeting effects of the AuNRs-PEG-NLS-RGD for the cell nucleus of the murine melanoma cell line (B16, having a smaller size of about 13 μm than HepG2 and SGC-7901), the dark-field and fluorescent images of cells were also taken. Under the dark-field irradiation, these nanoprobes produce strong scattering due to their plasmonic feature, while the cell nuclei had been stained with a nucleus-specific dye (Hoechst 33342, 10 μg/mL) that gives a blue color. It can be found from Figure 2g that most nanoprobes are distributed in the regions of the cell nuclei due to the dark-field/fluorescent merged images. Additionally, we compared three kinds of AuNRs including AuNRs-PEG, AuNRs-PEG-NLS, and AuNRs-PEG-RGD, incubating them with B16 cells, respectively, to verify the nuclear targeting feature of our nanoprobes, as shown in Figure S2. By comparing AuNRs-PEG-RGD and AuNRs-PEG-NLS, most of the AuNRs-PEG-NLS probes entered into the cell nucleus, which illustrates the nucleus-targeting ability of the NLS peptide. To further prove that the nanoprobes can enter the cell nucleus with the help of RGD and NLS, we labeled the NLS with a dye (fluorescein isothiocyanate, FITC) to display the locations of nanoprobes under the fluorescence microscopic imaging (IX71, Olympus) (Figure S3) and we measured the fluorescence spectrum of AuNRs-RGD-NLS (FITC), as shown in Figure S4. It can be observed that the fluorescence signal is still visible although the quenching exists. Besides, the fluorescent images of cells incubated with AuNRs-RGD-NLS (FITC) nanoprobes further prove their fluorescent activity. The green shows the distribution of nanoprobes, while the blue represents the cell nucleus. It can be seen that two colors overlap to a large extent, indicating that the nanoprobes have been delivered to the nucleus successfully. We further quantitated the amount of nanoprobes internalized into each B16 cell due to the plasmonic absorption of AuNRs *via* UV-vis spectroscopy, and the number is calculated as about 2,820 AuNRs per cell (see Part 2.5 in Supplementary Material).

### PDT-Dominating Treatment for B16 Cells

**Figure 3:**
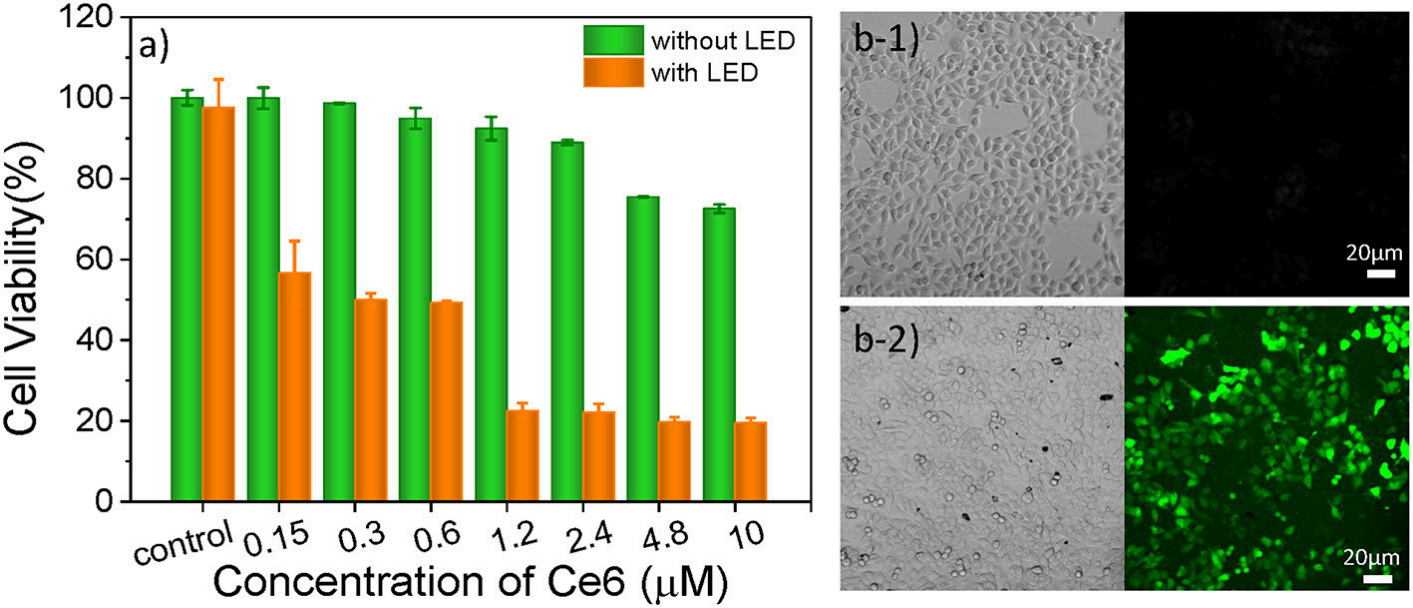
**(a)** Cell viabilities of B16 cells incubated with Ce6 under different concentrations for 12 h before and after the irradiation of a 650 nm lamp (18 mW/cm^2^) for 5 min. Bright-field and confocal fluorescent images of Ce6 (1.2 μM) treated B16 cells stained with DCFH-DA (10 μM) before **(b1)** and after **(b2)** they were treated with 650 nm LED (18 mW/cm^2^) for 1 min.

As one of three key components of PDT, PS endows the role of producing ROS. In this study, Ce6 is used as PS for treating B16 cells (Figure S5C), which is a porphyrin derivate (its structure is shown in Figure S5A). Ce6 has two strong absorption bands at 402 and 655 nm (Figure S5B). Thus, a 650 nm light-emitting diode (LED) array with a power density of 18 mW/cm^2^ (Figure S5D) was chosen for matching the long-wavelength band of Ce6. The cell culture time of Ce6 in B16 cells was evaluated as 12 h, which allows for the largest accumulation of Ce6 in cells (Figure S6). As shown in Figure 3a, as the concentration increased, there was no significant increase in toxicity without light irradiation, which means the Ce6 has negligible damage to B16 cells However, once they were exposed to the 650 nm light, cell viability decreases significantly with the Ce6 concentration increasing. When the treated concentration reaches 1.2 μM, the cell viability is only 22.5%. While, the survival rate of cell didn't further decrease even treated with higher dose. Therefore, the concentration of Ce6 for B16 cells is optimized as 1.2 μM by the WST-1 assay.

In order to verify that the photosensitizers (Ce6) can enter into the cancer cell and produce ROS under the irradiation of 650 nm LED, the 2, 7-dichlorofuorescin diacetate (DCFH-DA) probe that can monitor the generation of the intracellular ROS was used to evaluate the pharmaceutical effect of Ce6, owing to a fluorescent turn-on chromogenic reaction from DCFH-DA to dichlorofluorescein (DCF) after undergoing intracellular deacetylation upon ROS-mediated oxidation. Figure 3b shows the confocal fluorescent images of the DCFH-DA (10 μM) stained B16 cells that had been treated with Ce6 (1.2 μM) before and after they were exposed to a 650 nm lamp for 1 min. There is no obvious fluorescence signal before the light irradiation. After irradiating the cells for 1 min, obvious green fluorescence displays, proving the generation of ROS inside the PDT-treated cells (Figure 3b2). It can also be observed that many small bubbles appeared in cells and the morphology of cells became a nearly spherical shape compared those in Figure 3b1) that show a spindle shape, indicating the joint influence of the Ce6 (1.2 μM) and that the light irradiation (18 mW/cm^2^, 1 min) is enough for inducing these B16 cells dying.

**Figure 4:**
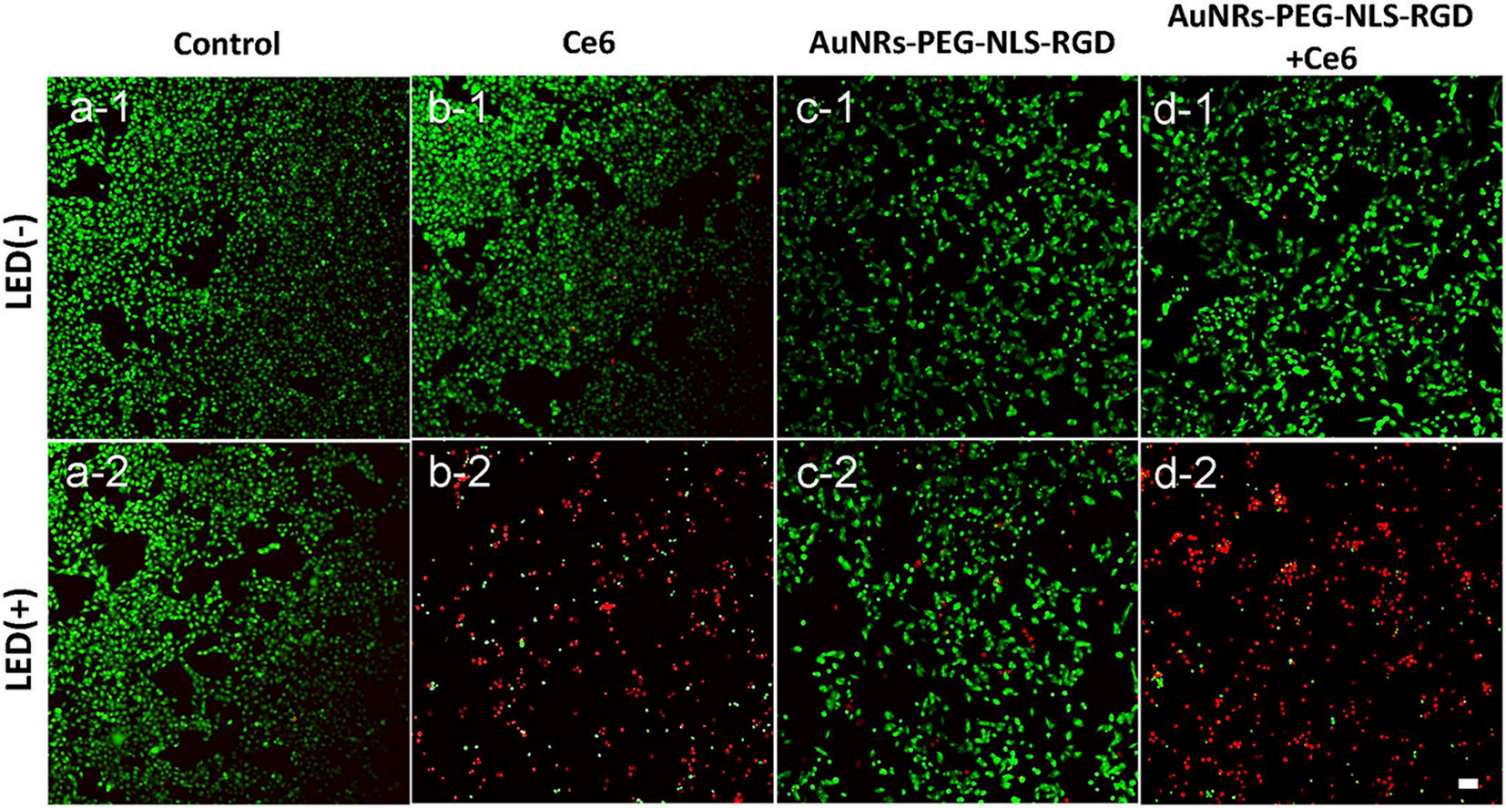
Confocal fluorescent images of B16 cells after they were incubated with Ce6 **(b)** and AuNRs-PEG-NLS-RGD **(c)** and AuNRs-PEG-NLS-RGD + Ce6 **(d)**, without (1) and with (2) the irradiation of a 650 nm lamp (18 mW/cm^2^) for 5 min, while the cells without and with the light irradiation were used as the control samples **(a)**. Cells were stained with Calcein-AM (green) and PI (red) for distinguishing the living and dead cells. The scale bar is 20 μm.

To further assess the effect of PDT treatment, confocal fluorescence microscopy was used to visually analyze cell viability with PDT treatment. These cells were stained with propidium iodide (PI, red) and Calcien-AM (green), respectively, which can distinguish dead and living cells. As shown in Figures 4a1,a2, the light can produce no damage to B16 cells. Similarly, if the cells were treated with Ce6 only, no dead cells were observed (Figure 4b1). When cells were exposed to Ce6 (a final concentration of 1.2 μM) and 650 nm light for 5 min (18 mW/cm^2^) simultaneously, we can find that a lot of cells were dead (Figure 4b2), which agrees with the results obtained by the WST-1 assay (Figure 3a). As we all know, AuNRs have good light-heat conversion efficiency and they will produce very high local temperature under the irradiation of light with a specific wavelength to induce cell death. Therefore, to identify whether there is a photothermal therapy (PTT) effect in our system, the images of B16 cells were cultured with the AuNRs-PEG-NLS-RGD nanoprobes (with a final concentration of 0.1 nM), and results with and without light exposure were obtained. The results show that AuNRs-PEG-NLS-RGD probes show no obvious toxicity to cells (Figure 4c1). Even after light irradiation, only a few dead cells were observed in Figure 4c2, indicating that cell death caused by the PTT can be ignored. Also, compared with PDT treatment (Figure 4b2), there is not much difference for cells treated with PDT and AuNRs-PEG-NLS-RGD probes at the same time, (Figures 4d1,d2) confirming that the therapeutic effect is dominated by the PDT in present system.

### Nuclear Dynamic Changes Revealed by SERS

**Figure 5:**
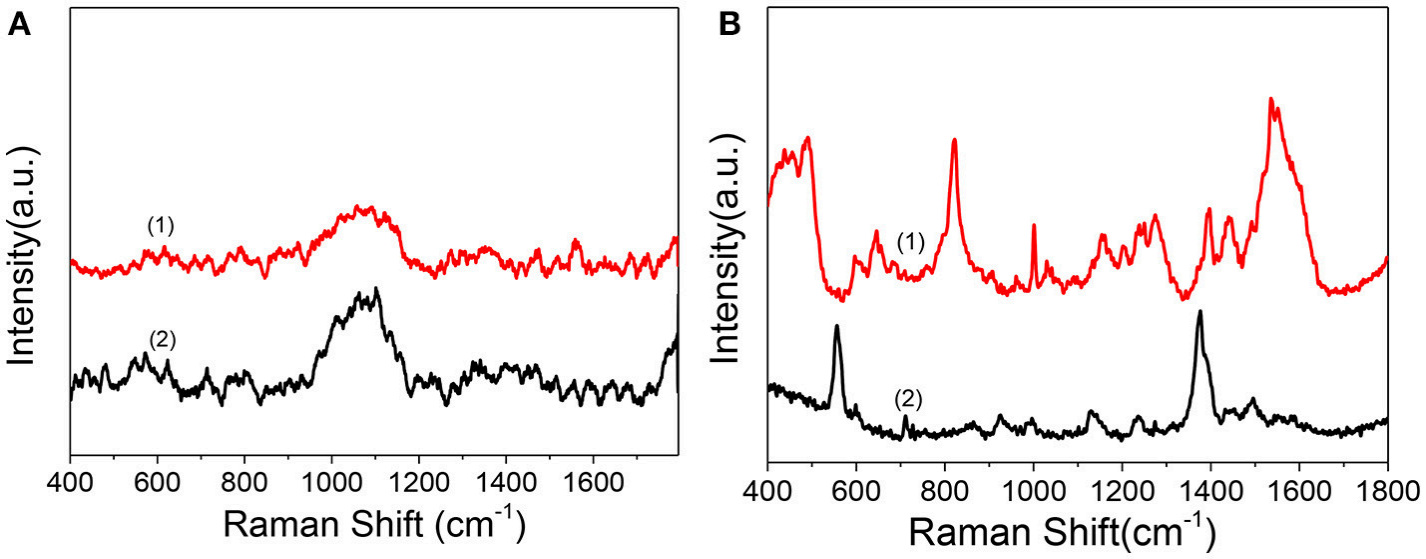
**(A)** Raman spectrum (1) and SERS spectrum of Ce6 solution (2) measured after mixing AuNRs (0.3 mL, 0.1 nM) with the Ce6 solution (0.3 mL, 1.2 μM). **(B)** (1) SERS spectrum of B16 cell nucleus with the enhancement of nuclear targeting nanoprobes (0.1 nM). (2) SERS spectrum of 1.0 nM of nuclear targeting nanoprobes.

As in the above discussion, we can obtain the therapeutic effect of the PDT treatment by WST-1 method. Confocal florescence imaging technique can also distinguish and analyze the dead and live cells visually by choosing the specific dyes. However, limited information during the dynamic treatment process can be known. So, we used SERS to reveal the molecular events of the cell nucleus during treatment. Before SERS detection of cells, to demonstrate whether there is obvious interference of the Ce6 on the SERS detection of the cell nucleus, we compared the Raman spectra and SERS spectra of Ce6 with SERS spectra of the nucleus. As shown in Figure 5A, by comparison, we can find that neither Raman spectra nor SERS spectra of Ce6 interferes with SERS detection of B16 cell nucleus.

In addition, to verify whether the SERS signals of targeted peptides on the nanoprobes would interfere in the results analysis of intranuclear components, we measured the SERS spectrum of the nuclear targeting probes (Figure 5B bottom curve). When the spectrum is compared with the nuclear SERS spectrum (Figure 5B top curve), despite several overlaps, most peaks are different, which indicates these nanoprobes have little interference in the spectral analysis of intranuclear biomolecules. This is consistent with our previous studies that the SERS spectra of the PEG, RGD, and NLS on the AuNRs are identifiable and distinguishable before and after they were in the cell nucleus (Liang et al., 2015; Deng et al., 2017; Shen et al., 2018).

**Figure 6:**
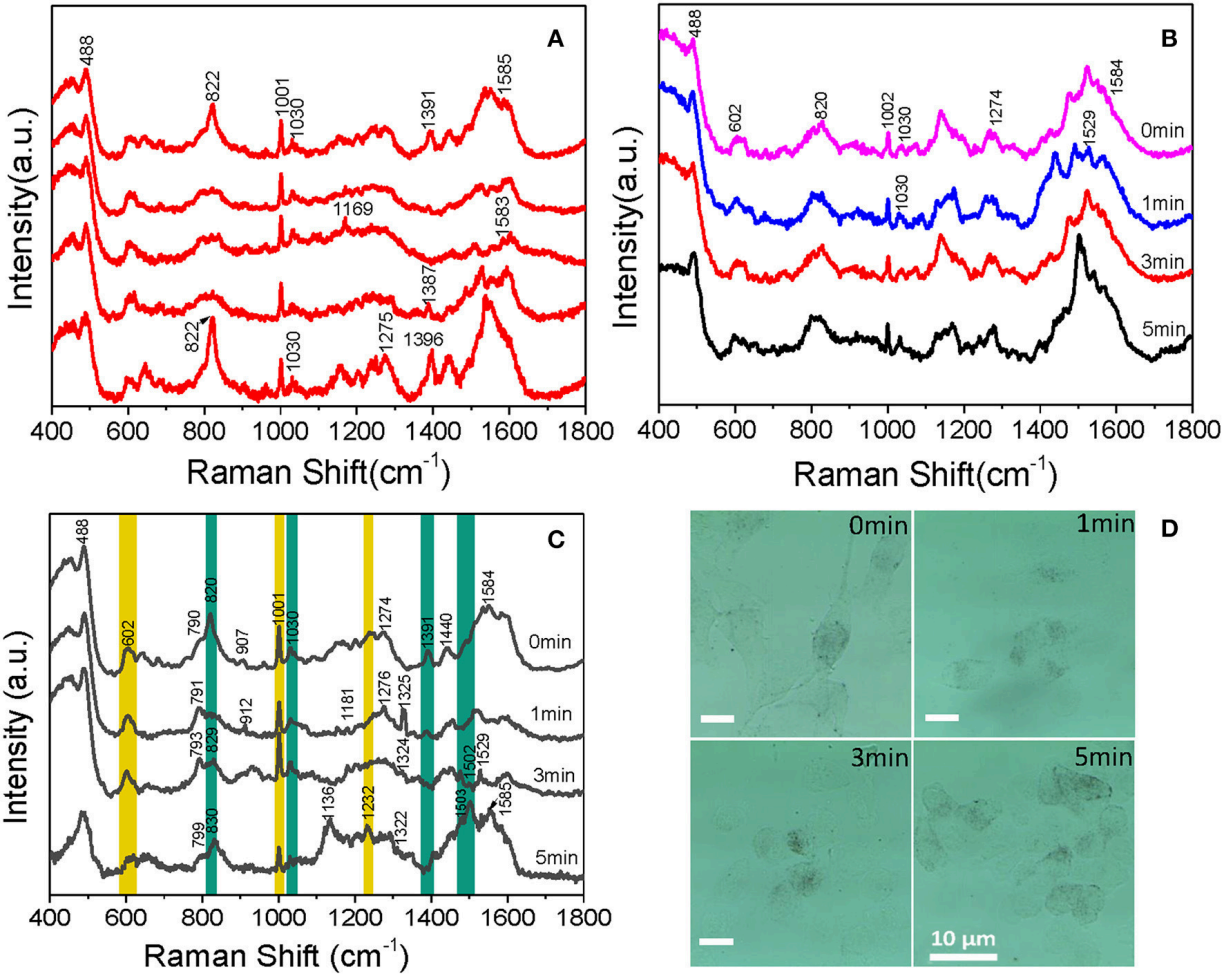
Intranuclear SERS spectra of **(A)** five B16 cells cultured with 0.1 nM AuNRs-PEG-NLS-RGD for 12 h, **(B)** cells cultured with 0.1 nM of AuNRs-PEG-NLS-RGD for 12 h and then treated with 650 nm LED (18 mW/cm^2^) for 0, 1, 3, and 5 min, respectively and **(C)** B16 cells cultured with 0.1 nM AuNRs-PEG-NLS-RGD and 1.2 μM Ce6 for 12 h and then treated with 650 nm LED (18 mW/cm^2^) for 0, 1, 3, and 5 min, respectively. **(D)** Bright-field images of B16 cells cultured with 0.1 nM AuNRs-PEG-NLS-RGD and 1.2 μM of Ce6 for 12 h and then treated with 650 nm LED (18 mW/cm^2^) for 0, 1, 3, and 5 min, respectively.

With the aim of understanding the therapeutic mechanism of PDT, we monitored the dynamics of intranuclear components by SERS. Before the experiment, we measured the SERS spectra of several proteins and DNA, such as bovine serum albumin (BSA) and calf thymus DNA (the data were not given). The spectra suggested that the signal we measured in B16 cells came from proteins and DNA rather than small molecules. Then, to assess the reproducibility of the obtained SERS spectra, the SERS spectra of B16 cells treated with the AuNRs-PEG-NLS-RGD nanoprobes were recorded. There are five intranuclear SERS spectra shown in Figure 6A, which demonstrates better reproducibility of the SERS spectra. Besides, the SERS spectra of the B16 cell nucleus, which were co-cultured only with the AuNRs-PEG-NLS-RGD nanoprobes and then irradiated with a 650 nm LED array (18 mW/cm^2^) for 0, 1, 3, and 5 min, respectively, were acquired (Figure 6B). Apparently, the characteristic peaks are almost unchanged with the increase of the illumination time. These results demonstrate that the molecular information of the cell nucleus has no obvious changes when the cells were only treated with nuclear-targeted nanoprobes or light if the PS is absent.

Afterward, B16 cells were incubated with nanoprobes and Ce6 in turn for 12 h each and then treated with a 650 nm LED array (18 mW/cm^2^) for the same time as mentioned above. The time-dependent SERS spectra and possible molecular events of the nuclear components were obtained, as shown in Figure 6C. These spectra were collected mainly from the definite spots where the nanoprobes were accumulated.

Each spectrum is an average spectrum of 150 spectra from six cells to reduce the differences between cells and provide convincing repeatability (Figure S7 shows the data of another batch). Table 1 summarizes the band assignments of the main components of the B16 cell nucleus in the SERS spectra of Figure 6C. The band at 602 cm^−1^ represents the -S-S- vibration of proteins. It can be seen from the spectrum that its intensity decreases with the increase of treatment time and reaches a minimum at 5 min, demonstrating the disruption of disulphide bonds and the denaturation of the protein tertiary structure. At the same time, we can see that the intensity of the Raman band at 1,001 cm^−1^, which is attributed to the respiration vibration of the phenylalanine in protein (Sau and Murphy, 2004) decreases at the irradiation time of 1 min, while it increases significantly at 3 min and arrives in the lowest after 5 min light irradiation. The apparent enhancement of peak intensity symbolizes the beginning of cell apoptosis (Ali et al., 2016). We hypothesize that the decrease of intensity may be due to the oxidation of phenylalanine to L-tyrosine under the action of ROS and enzyme, which further explains why the peak at 829 cm^−1^ (belonging to L-tyrosine) appears at 1 min light irradiation. Grune et al. (Kang et al., 2012) pointed out that amino acid residues, including tyrosine in proteins, are important reaction targets because of their active reactivity. They always become the primary targets of oxidative attacks on proteins. Thus, in the present study, the peak intensity at 829 cm^−1^ gradually increases with the increase of time as a result of PDT.

**Table 1:** The band assignment of the SERS spectra of the main components in B16 cell nucleus.

**Raman Shift(cm**^****−1****^**)**				**Assignments**		
**0 min**	**1 min**	**3 min**	**5 min**	**Carbohydrate**	**DNA**	**Protein**
488	488	488	488	Mannitose		
602	602	602	602			-S-S-
820	-	-	-		O-P-O stretch	
-	829	829	830			Tyrosine
907	912	-	-			C-C
1,001	1,001	1,001	1,001			Phenylalanine ring breath
1,030	1,031	1,032	1,030		C-O stretch	
-	1,181	1,180	1,181			C-N stretch
-	-	-	1,232			Amide III
1,391	1,390	-	-		O-P-O stretch	
-	-	1,502	1,503		A	
-	-	1,529	1,531		G	

Cellular biology discloses that when cells are stimulated by ROS, apoptosis inducing factor (AIF) is released from the mitochondria, then transferred into the cytoplasm and then into the nucleus, causing the DNA within the nucleus to agglutinate and break into fragments, inducing apoptosis (Grune et al., 2001). In the present study, at 5 min, a new band appears at 1,232 cm^−1^ (attributable to the C-N stretch, peptide bond), it is possible that the peptide bonds exposed to the surface of AuNRs. Bands at 820 and 1,391 cm^−1^, corresponding to O-P-O vibration of DNA backbone, gradually disappear with the increase of treatment time. This backbone structure leads the DNA base pairs exposing to the surface of AuNRs or closer to AuNRs, which is the reason why the intensities of the bands at 1,503 and 1,529 cm^−1^ belonging to the DNA base pair increase. Meanwhile, a decrease in the intensity of the band at 1,030 cm^−1^ is observed, which represents the C-O vibration in DNA, and further proves DNA fragmentation. The 1,529 cm^−1^ peak decreases at 5 min, which is mainly because guanine is the most easily oxidized base by singlet oxygen among the four bases of nucleic acid (Buchko et al., 1995). In addition to the orderly changes in peak intensities, Raman shifts of several bands also vary regularly. For example, peaks at 790 and 907 cm^−1^ shifts to higher wavenumbers. Since they represent DNA and protein, all these variations in Raman band intensity and shift suggest that DNA and protein in the nucleus of B16 cells has been destroyed and cell apoptosis occurs during the treatment of Ce6.

Beyond that, the process of Ce6 acting on B16 cells can also be researched by sustained cell deformation, a visualization process displayed on bright-field images (Figure 6D). Since B16 cells are adhere-wall cultured, we can see that there are many long filaments around individual cells and the shape of cells is fusiform before the treatment. After incubation with AuNRs-PEG-NLS-RGD, Ce6 and light irradiation, the morphology of B16 cells gradually shrank and eventually turned into a small spherical shape. At the same time, the adherence of cells gradually reduced, which caused them to float in the culture medium when they approached apoptosis. This visual process demonstrates that, with the prolongation of the action time of Ce6 in the body, Ce6 performs its damaging action gradually.

Apart from the SERS spectra of nucleus during PDT treatment processes, we investigated that the dynamic changes of intranuclear biomolecules treated with phorbol myristate acetate (PMA, 1 μg/mL), a membrane-permeable ROS generation stimulus (Bellavite, 1988), to further explore whether PMA will produce the same PDT effect as Ce6. SERS spectra of cell nuclei were recorded after they were treated with PMA for 1 and 2 h. As anticipated, we observed similar changes in peak intensities at 602, 820, 1,236, and 1,389 cm^−1^ (Figure S8). This observation is consistent with the above changes obtained from the treatment of Ce6 and confirms the therapeutic effect of the photosensitizer Ce6.

### PDT-Induced Apoptosis Revealed by Flow Cytometry

**Figure 7:**
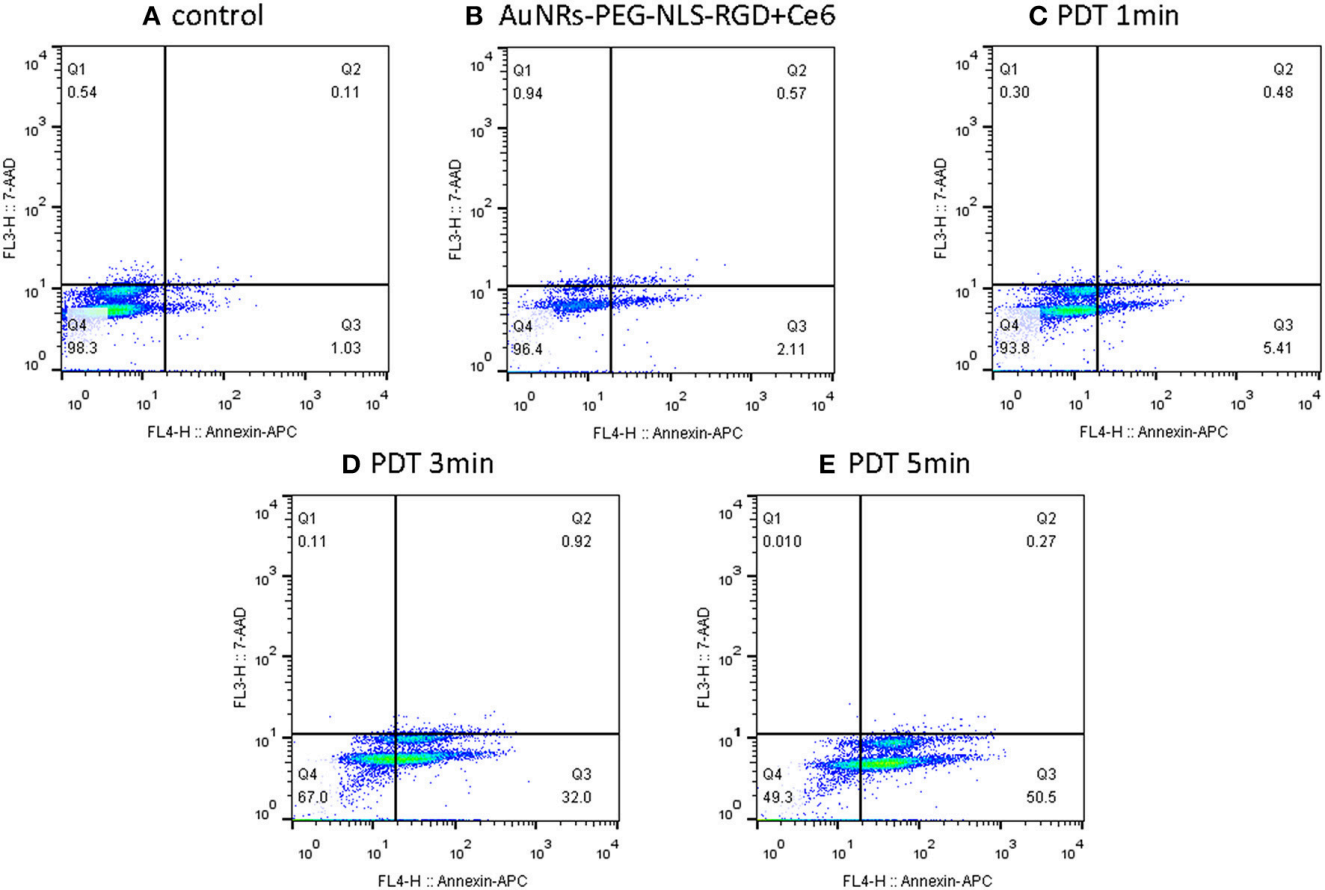
Flow cytometry (apoptosis/necrosis assay) for the B16 cells treated with nanoprobes and Ce6 before **(B)** and after light irradiation with different time **(C–E)**, while the B16 cells without any treatment was used as a control **(A)**. Cells were labeled with Annexin V-APC and 7-aminoactinomycinD (7-AAD) and measured by the FACSCalibur (BD Biosciences, USA). Q1- Q4 indicate necrosis, late apoptosis, early apoptosis and viable, respectively.

In addition to the SERS spectra, the effect of PDT was confirmed by the apoptosis/necrosis assay in which cells were labeled with Annexin V-APC and 7-aminoactinomycinD (7-AAD) and then the fluorescence intensity was examined by flow cytometry. It should be noted that the fluorescence emission bands of either Annexin V-APC or 7-AAD can effectively avoid the emission interference from Ce6. As shown in Figure 7, by comparing the control sample (B16 cells) and the B16 cells plus Ce6 before light irradiation, more and more apoptotic cells experiencing the PDT gradually appear in the Q3 phase that indicates an apoptosis-dominating physiological process, when the PDT strength increases as the irradiation time. These data well support the conclusion that the B16 cells undergo the way of apoptosis, which agrees with the above SERS results that the drastic changes of DNA and proteins in the cell nucleus were observed during this apoptotic process.

## Conclusions

SERS was successfully employed to monitor the dynamics of the murine melanoma cell nucleus during the PDT process. The nuclear targeted nanoprobes with significant SERS enhancement ability, specific targeting and excellent biocompatibility were designed and used for SERS measurements of the cell nucleus. With the assistance of targeting nanoprobes, the time-dependent SERS spectra along the course of the PDT treatment were achieved and the events of proteins and DNA molecules in the nucleus of cancer cells were disclosed. The apoptotic experience of a cell nucleus during the PDT was obtained and described. From this, we can conclude that the photosensitizer Ce6 may interfere with cell reproduction and induce cell apoptosis during PDT treatment, which has been proved by the data of flow cytometry. We believe that SERS tracing real-time cellular dynamic molecular changes of cancer cells in cell level during the PDT process will help with understanding the underlying molecular mechanisms in photodynamic cancer cell death.

## Author Contributions

JY, LL, YS, SX, CL, and WX: designed research; JY, XG, and ZL: performed research; JY, LL, JZ, YS, and RD: analyzed data; CL and WS: provided the cell culture conditions; JY, LL, and SX: wrote the paper. All authors have approved the final version of the manuscript.

### Conflict of Interest Statement

The authors declare that the research was conducted in the absence of any commercial or financial relationships that could be construed as a potential conflict of interest.
