# Supplementary material for: Investigating Dynamic Molecular Events in Melanoma Cell Nucleus During Photodynamic Therapy by SERS
Source: Front Chem. 2019 Jan 28;6:665. doi: 10.3389/fchem.2018.00665 (PMC6360157; doi:10.3389/fchem.2018.00665)
Supplement: Supplementary file 1 [file Presentation_1.pdf]

Supplementary Material

Investigating Dynamic Molecular Events in Melanoma Cell Nucleus during Photodynamic Therapy by SERS

Jing Yue[1], Lijia Liang[1], Yanting Shen[1], Xin Guan[2], Jing Zhang[1], Zhiyuan Li[3], Rong Deng[1], Shuping Xu[*1], Chongyang Liang[*2], Wei Shi[3] and Weiqing Xu[1]

*** Correspondence:** Shuping Xu: xusp@jlu.edu.cn (Xu S.); Chongyang Liang: liang@jlu.edu.cn (Lang C.)

1. Experimental Section

1.1 Materials and method

Tetrachloroauric acid trihydrate ($HAuCl_4 \cdot 3H_2O$) (Aladdin Industrial Corporation), cetyltrimethylammonium bromide (CTAB) (Aladdin Industrial Corporation), Ascorbic acid(Beijing Chemical Company), $AgNO_3$(Shanghai Chemical Company), $NaBH_4$(Tianjin Fuchen Chemical Reagents Factory), Methoxypoly(ethylene glycol)-thiol (mPEG-SH, MW=5000) (Tianjin Red Sun Jin Boda Biological Technology Co., LTD), Cell membrane penetrating peptide (RGD, RGDRGDRGDRGDPGC), nucleus targeting peptide (NLS, CGGGPKKKRKVGG), fluorescein isothiocyanate (FITC)-labeled NLS (NLS-FITC) were both purchased from Apeptide (Shanghai). 2',7'-dichloro-fluorescein diacetate (DCFH-DA, Sigma) Roswell Park Memorial Institute 1640 medium (RPMI 1640, Invitrogen), Hoechst 33342 (Invitrogen), phorbol myristate acetate (PMA, Sigma) and Chlorin e6 (Ce6, Sigma).

We employed JEM-2100F field emission transmission electron microscope (TEM, JEOL, Tokyo, Japan) to characterize the morphology of the prepared AuNRs. Ultraviolet-visible (UV–vis) spectroscopy (Ocean Optics, USB4000) and dynamic light scattering (DLS, Malvern Zetasizer Nano ZS) were used to characterize the fabrication of the nanoprobes. And we detected the specific targeting effects of AuNRs-PEG-RGD-NLS by self-built detection platform integrated with both fluorescence microscope (IX71, Olympus) and dark-field microscope (Olympus). SERS spectra were performed on a confocal Raman system (LabRAM Aramis, Horiba JobinYvon, USA) with a 785 nm laser as the excitation source. We used self-built 650nm LED lamp (a Petri dish was placed inside a box with the LED, the distance between LED and the Petri dish is about 25cm.The power at the position of Petri dish is about $18 mW/cm^{-2}$.) to produce a therapeutic effect. FV1000 confocal fluorescence microscope (Olympus) was employed to identify the location of Ce6 and prove that AuNRs had a weak PTT effect on the cells under such lamp irradiation. For flow cytometry analysis, the fluorescence intensity was recorded by the FACSCalibur (BD Biosciences, USA).

1.2 Synthesis of gold nanorods (AuNRs)

Here, AuNRs which were stabilized by cetyltrimethylammonium bromide (CTAB) were synthesized according to the modified seed-mediated growth method. 120 μL of 15mM $HAuCl_4$ was added to the 5.0 mL of a 0.2 M CTAB solution, then 500 μL 0.01 M of ice-cold $NaBH_4$ was added to the mixture under stirring for 2 min. The mixture was aging 2.5 hrs at the temperature of 30 °C to

obtain the Au seeds. Then 500 µL of the seed solution was added to a mixture containing 44.38 mL of a 1.0 M CTAB aqueous solution, 2.5 mL of 15 mM $HAuCl_4$, 2mL of 4.0 mM $AgNO_3$, and 620 µL of 78.8 mM ascorbic acid. The mixture was kept undisturbed for 12 h at 28°C.

1.3 Fabrication of nucleus-targeting nanoprobes

AuNRs were modified by mPEG-SH, NLS, and RGD in one step. 150 µL of mPEG-SH solution (0.01 mM), 2.4 µL of RGD (5.0 mM) and 12 µL of NLS (5.0 mM) aqueous solutions were added to 10.0 mL of AuNR solution and then the mixture was allowed to react for 24 h at room temperature. Excess mPEG-SH, NLS and RGD were removed by centrifugation (5000 rpm, 10 min) to achieve the nuclear targeted probes (AuNRs-PEG-RGD-NLS) after 24h. They were redisposed in 1.0 mL deionized water before use. The molar ratio of RGD with AuNRs is 10^3:1, whereas the molar ratio of NLS is 10^4:1.

2 Supplementary FiguresQuantification of the NLS and RGD peptides on single AuNR

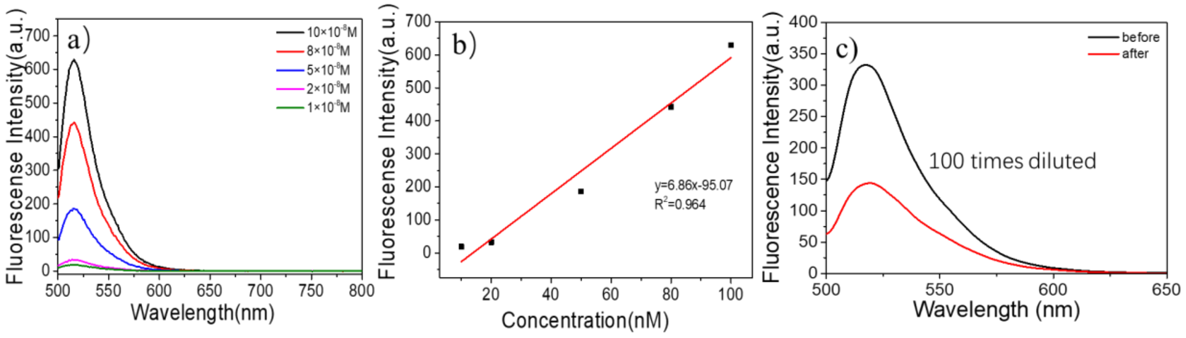

Supplementary Figure 1. Fluorescence spectra of the FITC FITC-labelled NLS a) with different concentrations. Calibration curve of that which was is obtained by plotting the fluorescence intensity at 516 nm against the concentration of the FITC-labelled NLS peptide b). The averaged fluorescence intensity of c) the free FITC-NLS plus RGD before and after reacting with AuNRs (2.7 nM, 1.0 mL). The solutions for c) is 100 times diluted.

2.2 Location of three nanoprobes in cells

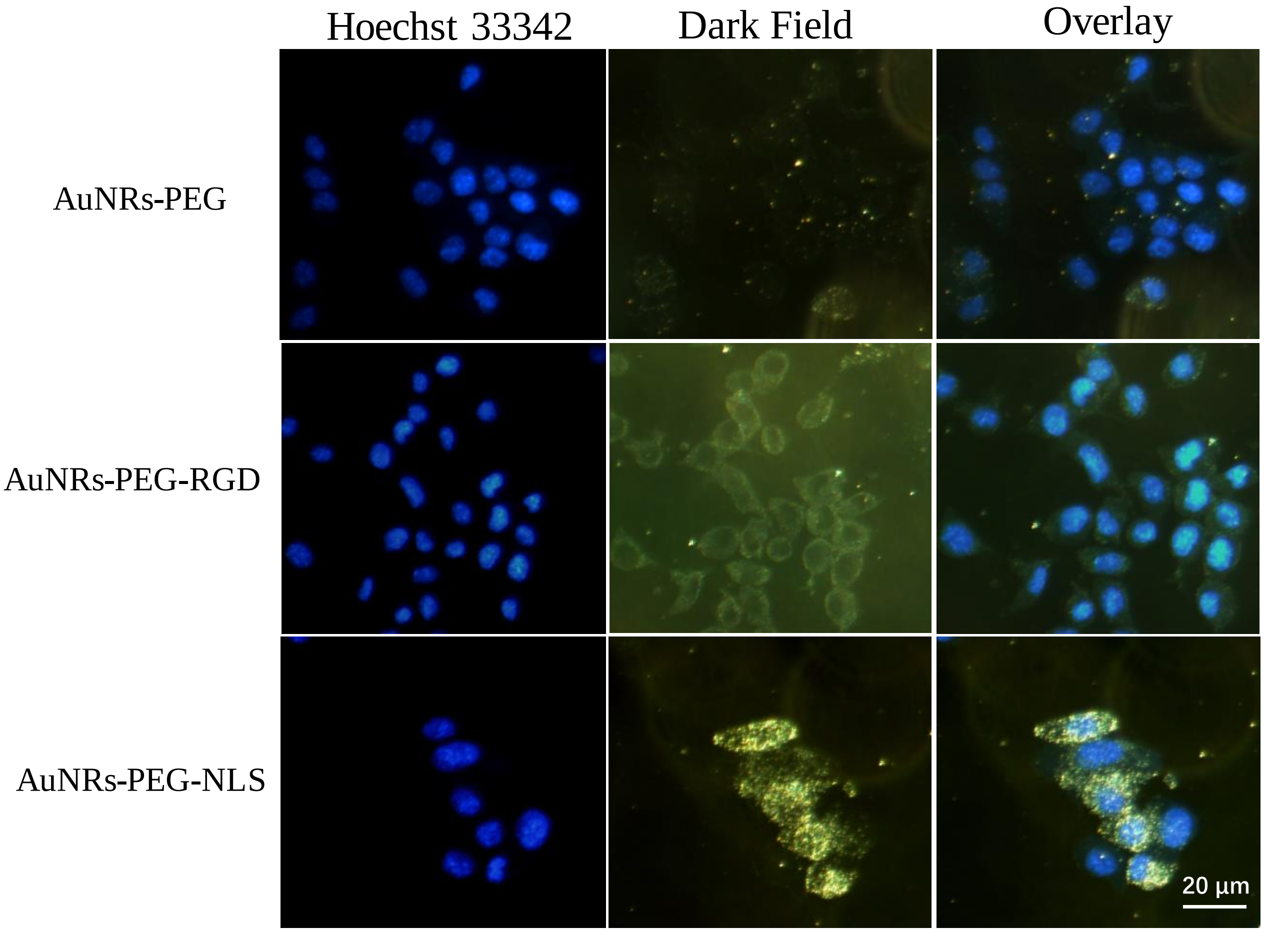

Supplementary Figure 2. Fluorescent, dark-field, and overlay images of B16 cells incubated with AuNRs-PEG, AuNRs-PEG-RGD and AuNRs-PEG-NLS for 12 h from top to bottom.

2.3 Location of nanoprobes in nucleus

To take the confocal fluorescent images of nanoprobes to ensure the ability of targeting, the FITC-labeled targeted nanoprobes were prepared. The FITC-labeled NLS were used to modify the surface of AuNRs instead of the pure NLS to obtain the FITC-labeled AuNRs-PEG-RGD-NLS. Besides this, the self-built detection platform integrated with both fluorescence microscope (IX71, Olympus) and dark-field microscope (Olympus) were also employed.

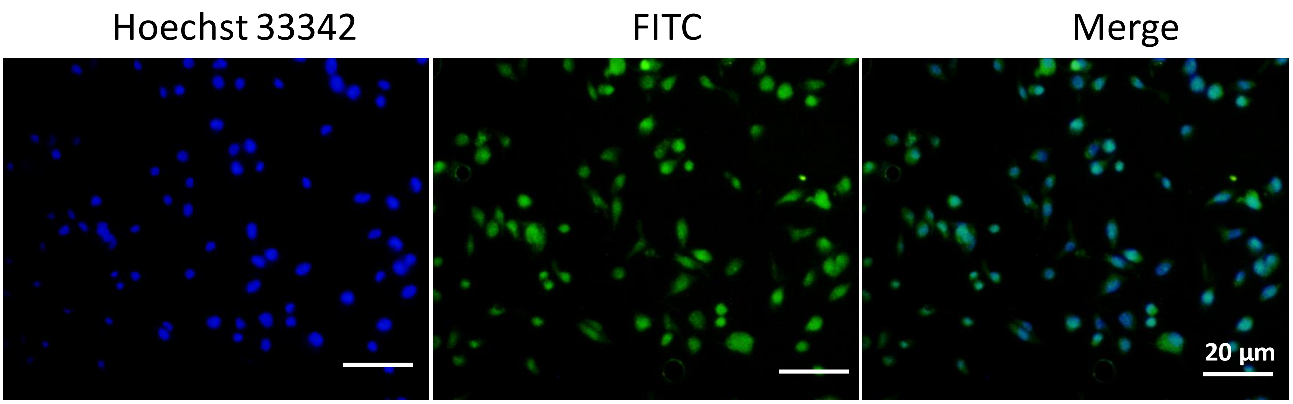

Supplementary Figure 3. Fluorescent and merged images of B16 cells cultured with FITC-labelled nucleus targeting nanoprobes for 12 h. And the nucleus was stained with blue by Hoechst 33342.

2.4 Fluorescence spectra of FITC-NLS and AuNRs-RGD-NLS(FITC)

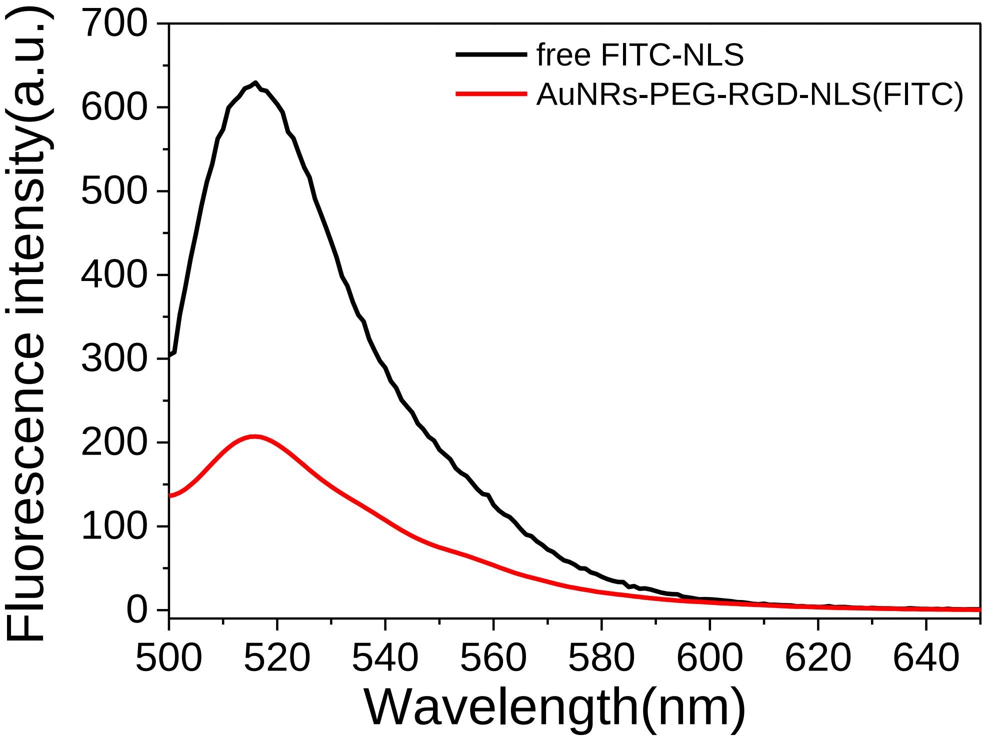

Supplementary Figure 4. The averaged fluorescence intensity of the free FITC-NLS and AuNRs-PEG-RGD-NLS (FITC) (the solution was 50 times diluted).

2.5 Quantification of nanoprobes in cells

In order to quantify the internalization of the nanoprobe into cells, we calculated the amount of AuNRs-PEG-RGD-NLS nanoprobe in cell culture medium before and after incubation with cells via UV-vis spectroscopy based on the plasmonic feature of AuNRs. We co-culture the AuNRs-PEG-RGD-NLS nanoprobe (1.0 nM, 0.6 mL) with B16 cells for 12 h. After that, the culture medium

containing uninternalized nanoprobes was collected and centrifuged (6600 rpm, 6 min) to determine the amount of nanoprobes not uptake by the cells (0.3 nM, 1 mL). Therefore, the amount of the nanoprobes that were internalized is 2.102×10^{-13} mol, which means there are 1.27×10^{11} nanoprobes that entered into all cells (the density is about 4.5×10^{6}). Based on this, we evaluate that about 2820 nanoprobes were internalized or attached on per B16 cell.

2.6 Characterization of Ce6 and LED

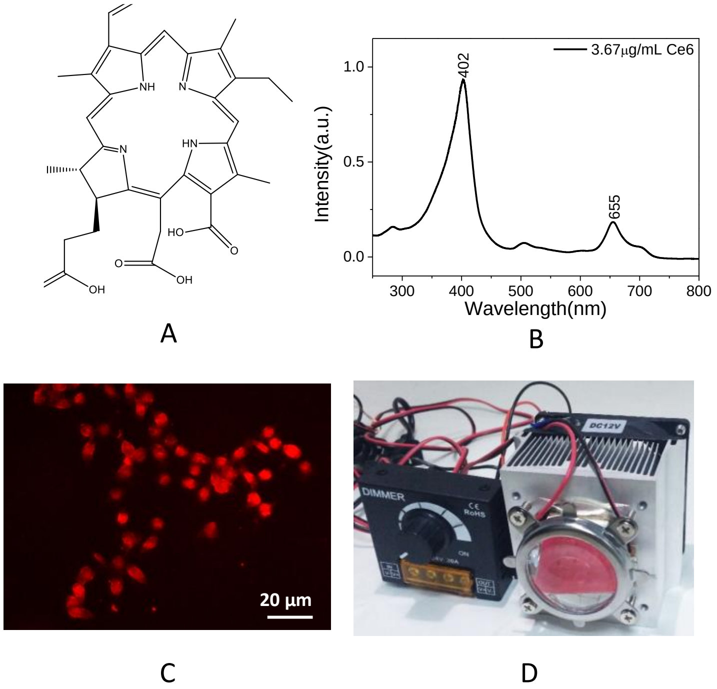

Supplementary Figure 5. The structure (A) and UV-vis spectra (B) of Ce6. (C) Fluorescent image of B16 cells cultured with 1.2μM Ce6 for 12h. (D) The LED lamp with a wavelength of 650 nm and a powder density of 18 mW/cm^2 used for the PDT study.

2.7 Internalization of Ce6

We co-cultured 1.2 μM of Ce6 with B16 cells for different times respectively to determine the proper culture time with the help of fluorescence microscopy. We can easily identify the location of Ce6 because of its red fluorescent emission (Figure S4). It can be observed that with the increase of the incubation time, the fluorescence intensity gradually increased and then decreased. Since the highest

fluorescence is observed when the incubation time is 12 h, indicating a largest accumulation of Ce6. So, we keep a culture time of 12 h for Ce6 in the following experiments.

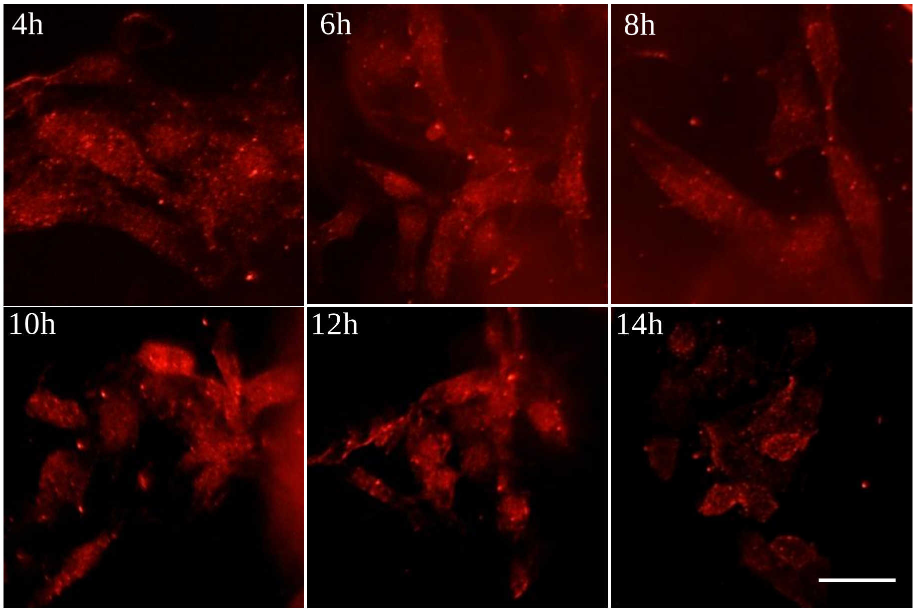

Supplementary Figure 6. Fluorescence images of B16 cells incubated with 1.2μM Ce6 for 2h,4h,6h,8h,10h,12h and 14h respectively. The scale bar is 10 μm.

2.8 Intranuclear SERS spectra of B16 cells at different treatment time

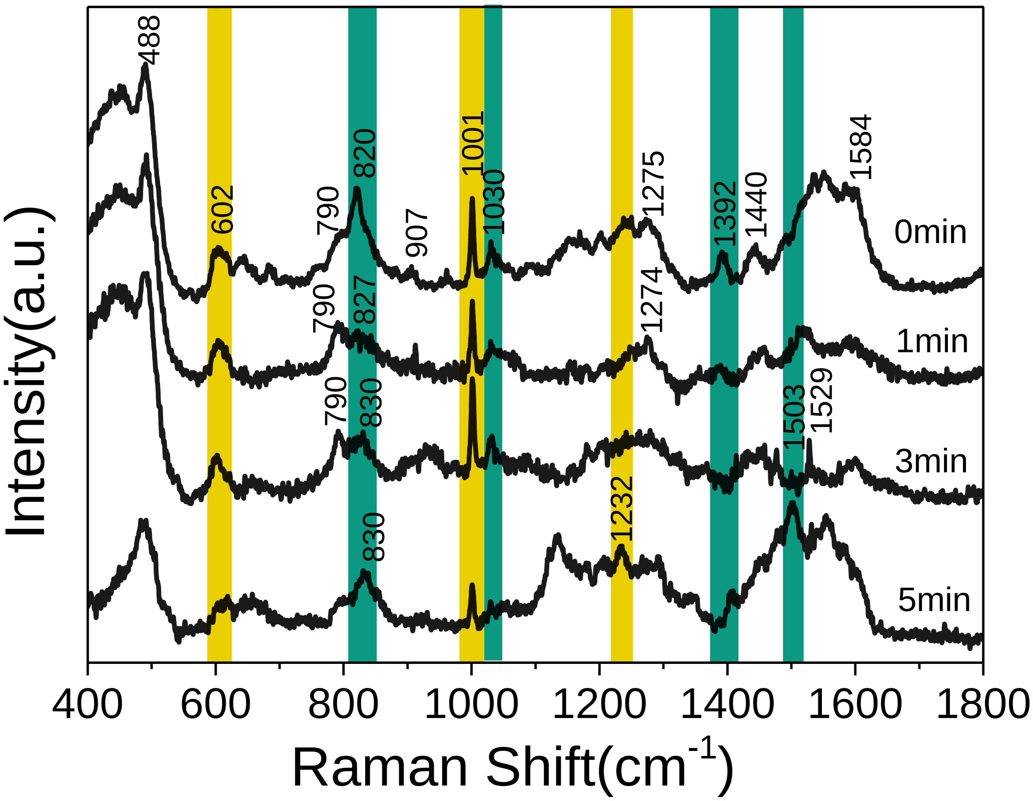

Supplementary Figure 7. Intranuclear SERS spectra of B16 cells cultured with 0.1 nM AuNRs-PEG-NLS-RGD and 1.2 µM of Ce6 for 12 h and then treated with 650 nm LED (18 mW/cm^2) for 0, 1, 3, and 5 min, respectively. These spectra are from another batch of cells.

2.9 Intranuclear SERS spectra of B16 cells treated with PMA

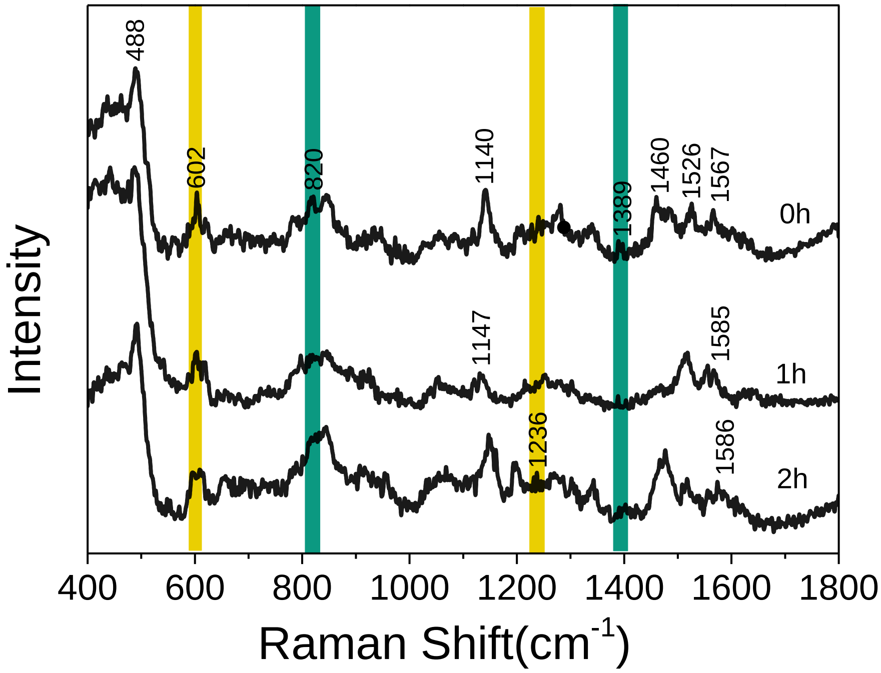

Supplementary Figure 8. Intranuclear SERS spectra of B16 cells cultured with 0.1 nM AuNRs-PEG-NLS-RGD after treated with PMA for 1, 2 h respectively.
